# Insights into ecological roles of uncultivated bacteria in Katase hot spring sediment from long-read metagenomics

**DOI:** 10.3389/fmicb.2022.1045931

**Published:** 2022-11-03

**Authors:** Shingo Kato, Sachiko Masuda, Arisa Shibata, Ken Shirasu, Moriya Ohkuma

**Affiliations:** ^1^Japan Collection of Microorganisms, RIKEN BioResource Research Center, Tsukuba, Japan; ^2^Plant Immunity Research Group, RIKEN Center for Sustainable Resource Science, Yokohama, Japan

**Keywords:** metagenomics, long-read sequencing, hot spring, thermophiles, uncultivated prokaryotes, microbial dark matter

## Abstract

Diverse yet-uncultivated bacteria and archaea, i.e., microbial dark matter, are present in terrestrial hot spring environments. Numerous metagenome-assembled genomes (MAGs) of these uncultivated prokaryotes by short-read metagenomics have been reported so far, suggesting their metabolic potential. However, more reliable MAGs, i.e., circularized complete MAGs (cMAGs), have been rarely reported from hot spring environments. Here, we report 61 high-quality (HQ)-MAGs, including 14 cMAGs, of diverse uncultivated bacteria and archaea retrieved from hot spring sediment (52°C, pH 7.2) by highly accurate long-read sequencing using PacBio Sequel II. The HQ MAGs were affiliated with one archaeal and 13 bacterial phyla. Notably, nine of the 14 cMAGs were the first reported cMAGs for the family- to class-level clades that these cMAGs belonged to. The genome information suggests that the bacteria represented by MAGs play a significant role in the biogeochemical cycling of carbon, nitrogen, iron, and sulfur at this site. In particular, the genome analysis of six HQ MAGs including two cMAGs of *Armatimonadota*, of which members are frequently abundant in hot spring environments, predicts that they are aerobic, moderate thermophilic chemoorganoheterotrophs, and potentially oxidize and/or reduce iron. This prediction is consistent with the environmental conditions where they were detected. Our results expand the knowledge regarding the ecological potential of uncultivated bacteria in moderately-high-temperature environments.

## Introduction

Since the early 1990s, culture-independent molecular analyses targeting the 16S rRNA gene have revealed the presence of phylogenetically diverse uncultivated bacteria and archaea, i.e., microbial dark matter, in terrestrial hot springs (e.g., Barns et al., 1994; Hugenholtz et al., 1998; Takai and Sako, 1999; Kato et al., 2011). Some of these uncultivated prokaryotes have been classified into higher-rank clades at class- or phylum-level, including Obsidian Pool (OP) groups for bacteria (Hugenholtz et al., 1998), *Korarchaeota* (Barns et al., 1996), and Terrestrial Hot Spring Creanarchaeotic Group (THSCG; Takai and Sako, 1999) for archaea. Until now, several species of the clades have been isolated and physiologically characterized, for instance, *Armatimonas rosea* in the phylum *Armatimonadota* (or *Armatimonadetes*; formerly OP10; Tamaki et al., 2011), *Caldisericum exile* in the phylum *Caldisericota* (or *Caldiserica*; formerly OP5; Mori et al., 2008, 2009), and *Conexivisphaera calida* in the class *Conexivisphaeria* (formerly THSCG; Kato et al., 2019, 2021). However, the majority of the reported higher-rank clades have not contained cultivated representatives yet. Recent technological advances in metagenomics and single-cell genomics enable us to reconstruct nearly-complete genomes directly from environmental samples without cultivation. Indeed, numerous metagenome-assembled genomes (MAGs) and single-cell amplified genomes (SAGs) have been reconstructed from a variety of terrestrial hot spring environments, providing an important clue to understanding the ecological significance of uncultivated members for biogeochemical cycling in high-temperature environments (Nunoura et al., 2011; Rinke et al., 2013; Beam et al., 2014; Jarett et al., 2018; Ward et al., 2019).

In recent years, short-read sequencing (several hundreds of base pairs in read length) has been widely used to reconstruct MAGs. One limitation of MAG reconstruction from short reads is that it is difficult to construct circularized complete MAGs (cMAGs) due to repeated sequences (e.g., CRISPR regions) and/or multiple copies of long genes and operons, which are almost identical to each other (e.g., ribosomal RNA genes). Another concern is that, even if the MAGs are defined as high-quality (HQ) MAGs based on the minimal standards (Bowers et al., 2017), in some cases, MAGs from short reads consist of hundreds of contigs, which could increase the risk of undetectable contamination. In contrast, the hybrid assembly of short reads and long reads from complex microbial communities in natural environments has produced more reliable MAGs including cMAGs (Moss et al., 2020; Singleton et al., 2021). Moreover, highly accurate long-read sequencing, i.e., high-fidelity (HiFi) or circular consensus sequencing (CCS) by PacBio Sequel II, enables one to reconstruct reliable MAGs including cMAGs without short-read hybrid assembling (Hon et al., 2020; Bickhart et al., 2022).

Here, we report 61 HQ MAGs, including 14 cMAGs, reconstructed from a hot spring environment by PacBio HiFi sequencing. Based on the genome information of the HQ MAGs, we predict the metabolic potential of bacteria and archaea represented by the HQ MAGs, and discuss their ecological roles. In particular, we focus on the MAGs belonging to the phylum *Armatimonadota,* of which most members are yet-uncultivated. The metabolic potential of the uncultivated members of *Armatimonadota* has been poorly understood so far, despite the fact that they have been often detected, and abundant in some cases, in hot spring environments (Lee et al., 2014).

## Materials and methods

### Field description and sampling

Hot spring water (84.5°C, pH 6.7, 0.55% salinity, 0.0 mg/L of dissolved oxygen) discharging into a natural pool (Supplementary Figure S1) was observed in Katase hot spring field (34.804°N 139.064°E), Shizuoka, Japan. The water depth of the pool was 2–5 cm. Green microbial mats were observed on the surface of the bottom sediment. A sediment sample (called KatS3) was collected at the bottom of the pool (52.1°C, pH 7.2, <0.1% salinity, 3.85 mg/L of dissolved oxygen) on June 2012. The sediment sample was collected from 0 to 5 cm depth from the sediment surface using a sterilized spatula, transferred into sterilized plastic tubes, and stored with ice packs in a cooler box. The collected sample was taken into our laboratory within a day, and stored at −80°C until DNA extraction. The temperature, pH, the concentrations of dissolved oxygen and ferrous iron (Fe^2+^) of the hot spring water in the pool were measured as previously described (Kato et al., 2013). Dissolved sulfide (S^2−^) concentration was measured using a Gastec test tube (no. 211; GASTEC corp., Kanagawa, Japan). The concentrations of Fe^2+^ and S^2−^ in the hot spring water were under the detection limits (<18 and < 16 μM, respectively).

### DNA extraction and sequencing

DNA was extracted from the bulk sediment sample (3.3 g) using a FastDNA spin kit for soil (MP Biomedicals; Irvine, CA, United States). The extracted DNA was purified and concentrated using NucleoBond HMW DNA (Takara Bio; Kusatsu, Shiga, Japan). The fragmentation level of the purified DNA was checked by pulsed-field capillary electrophoresis using Femto Pulse System (Agilent Technologies; Santa Clara, CA, United States). A SMRTbell library was prepared using the HMW DNA by SMRTbell Template Prep Kit v.2.0 (Pacific Biosciences; Menlo Park, CA, United States), and was size-selected on the BluePippin system using a 0.75% agarose cassette (Sage Science; Beverly, MA, United States) and a 5–30 kb high-pass cutoff. The size-selected SMRTbell library was bound to the sequencing polymerase enzyme using the Sequel II Binding Kit 2.1. Shotgun genomic DNA sequence data were collected on one run (with one SMRT Cell) of the PacBio Sequel II system with HiFi sequencing protocols and Sequencing Kit 2.0 chemistry (PacBio). HiFi reads (or CCS reads) were generated using ccs software v.10.0^1^ with the default parameters (--minPasses 10 bp, --minPredictedAccuracy 0.0, and --maxLength 50,000 bp) and extracted >Q20.

### Counting and taxonomic classification of marker genes in HiFi reads

Sequences of taxonomic marker genes, i.e., 16S rRNA gene, and *rpsB* (for ribosomal protein S2) and *rplC* (ribosomal protein L3) as highly conserved genes, were directly extracted from the HiFi reads, and analyzed as follows. For 16S rRNA genes, we used the Perl script, get_ssu_for_genome_bin_tools.pl., included in gbtools (Seah and Gruber-Vodicka, 2015), of which taxonomic classification was based on Silva database release 138 (Quast et al., 2013). For *rpsB* and *rplC*, we used GraftM version 0.13.1 (Boyd et al., 2018) to count reads and their taxonomic classification.

### Construction and characterization of metagenome-assembled genomes

The HiFi reads were assembled by metaFlye version 2.8.3 (Kolmogorov et al., 2020), and were mapped on the generated contigs using bbmap version 38.34 (Bushnell, 2014). Initial bins were generated using the contigs and mapping data by MetaBat version 2.15 with -m 5,000 -x 5 --saveCls, and also by MaxBin version 2.2.7 with -min_contig_length 5000. Then, the initial bins were refined using the “bin_refinement” tool of MetaWRAP version 1.2.1 (Uritskiy et al., 2018) with -c 20 -x 10. The refined bins were used as MAGs for further analyses.

The MAGs were annotated using DFAST version 1.2.13 (Tanizawa et al., 2018) with Prodigal (Hyatt et al., 2010) for prediction of protein-coding regions (CDSs), tRNAscan-SE (Chan et al., 2021) for identification of tRNA genes, and Barrnap^2^ for identification of rRNA genes. Values of the average amino acid identity (AAI) among MAGs were calculated using EzAAI version 1.1 (Kim et al., 2021). Taxonomic classification of the MAGs was performed using Genome Taxonomy Database (GTDB)-tk version 2.1.1 (Chaumeil et al., 2019) with the R207 database. Prediction of optimum growth temperature from the MAGs was performed using Tome version 1.0 (Li G. et al., 2019). Functional annotation for CDSs was performed using METABOLIC version 4.0 (Zhou et al., 2022), FeGenie version 1.0 (Garber et al., 2020), and DiSCo version 1.0 (Neukirchen and Sousa, 2021), in addition to the KEGG mapper tool with GhostKOALA (Kanehisa et al., 2016). CDSs for putative c-type cytochrome (Cyc) proteins with one or more Cys-X-X-Cys-His (CXXCH) motifs were manually extracted. Subcellular localization of proteins were predicted using PSORTb version 3.0.2 (Yu et al., 2010). Clustering of protein sequences was performed using cd-hit version 4.7 with a 99% identity threshold (Li and Godzik, 2006). All the above analyses were performed using the default settings unless specified.

### Detection of viruses/phages and plasmids

Viruses/phages in all contigs were detected using VirSorter version 2.2.3 (Guo et al., 2021) and CheckV version 0.7.0 (Nayfach et al., 2021). Plasmids in all contigs were detected using MOB-typer version 3.0.2, a tool of MOB-suite (Robertson and Nash, 2018). All the above analyses were performed using the default settings unless specified.

### Phylogenetic analysis

To construct a phylogenetic tree of 16S rRNA genes for *Armatimonadota*, the nucleotide sequences in the MAGs were aligned with reference sequences using SINA version 1.2.11 (Pruesse et al., 2012) on the Silva website (Quast et al., 2013). The alignment was trimmed using TrimAl version 1.2 (Capella-Gutierrez et al., 2009) with the “-automated1” option. A maximum likelihood (ML) tree was constructed using IQ-TREE version 2.1.2 (Minh et al., 2020) with the GTR + F + I + G model. To construct a phylogenomic tree for *Armatimonadota*, the concatenated alignment of 120 marker proteins provided from GTDB-Tk was trimmed using TrimAl with the “-automated1” option, and used for ML tree construction using IQ-TREE with the LG + I + G4 model. To construct phylogenetic trees of protein sequences for CDSs, the alignments were generated using Muscle version 3.8.31, and then trimmed and used for ML tree construction as described above. Bootstrap support values were computed with 1,000 replicates for all trees.

### 16S rRNA gene survey in public databases

The 16S rRNA genes closely related to those of our *Armatimonadota* MAGs were surveyed in Sequence Read Archive (SRA) in National Center for Biotechnology Information (NCBI) using IMNGS (Lagkouvardos et al., 2016) with a 95% similarity threshold on May 2022.

## Results and discussion

### HiFi reads and assembly

The PacBio CCS resulted in 2,694,800 HiFi reads (27.96 Gbp) with an N50 of 10,544 bp. The longest read was 43,453 bp. Assembling of the HiFi reads resulted in 13,365 contigs with an N50 of 231,997 bp (39365.5 bp on average), of which the assembly graph is shown in Supplementary Figure S2. Of the total contigs, 554 were circularized, and up to 5.6 Mbp in length (Supplementary Table S1). The 5.6 Mbp circular contig was the longest among all the 13,365 contigs. Length, coverage, and GC content of the linear and circular contigs are plotted in Supplementary Figure S3. One notable feature is the two peaks at around 6 × 10^3^ bp and around 3 × 10^4^ bp of the length of the circular contigs. Another feature is a concavity at around 45–48% of GC content for the linear contigs. However, at present, their biological and ecological meanings are unclear.

Of the 554 circular contigs, 14 contigs including the longest contig were identified as prokaryotic genomes (Supplementary Figure S4), as described below in detail. The other 75 circular contigs were binned into MAGs with other contigs, which are potentially chromids (Harrison et al., 2010). It should be noted that 48 of the 75 chromid-like contigs were also potentially viruses/phages (Supplementary Table S1), suggesting that these are extrachromosomal prophages (Roux et al., 2015). No circular plasmid was detected in the assembly, although putative plasmids were identified in seven linear contigs (Supplementary Table S2). Three of the circular, short (<81.2 kbp), non-binned contigs possessing small subunit (SSU) rRNA genes were detected (contig_17641, contig_18268, and contig_18742). Each of the SSU rRNA sequences was most closely related to each of the following cultivated species, *Petalomonas acorensis* (*Eukaryota*) with an 85.7% similarity, *Lujinxingia vulgaris* (*Deltaproteobacteria*) with an 88.4% similarity, or *Anderseniella baltica* (*Alphaproteobacteria*) with a 77.3% similarity, respectively. It remains unclear if these originate from symbionts/organelles. The other 179 circular contigs were identified as potential viruses/phages. The origins of the remaining 283 circular contigs were unknown, of which length and number of CDSs were up to 193 and 228 kbp, respectively.

### Microbial community structure

**Figure 1:**
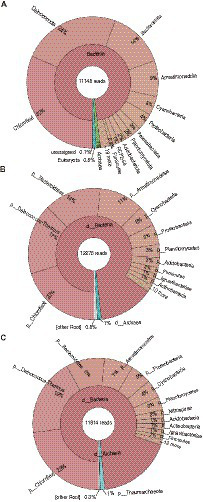
Microbial community structure. **(A)** 16S rRNA gene, **(B)** *rpsB*, and **(C)** *rplC*, were used for taxonomic profiling. The taxonomic names displayed in this figure were originally provided from each analytical tool [i.e., Silva database (Quast et al., 2013) for **(A)**, or GraftM packages (Boyd et al., 2018) for **(B,C)**].

To assess the microbial community structure in the metagenome, we analyzed two of highly conserved marker genes (i.e., *rpsB* and *rplC*) and the 16S rRNA gene in the HiFi reads. The results of taxonomic profiling of the extracted genes are summarized in Figure 1. Overall, the abundant taxonomic groups in the community were consistent in 16S rRNA and highly conserved marker gene analyses, even though the result of 16S rRNA gene analysis could be biased due to its copy numbers in genomes. In this community, members of the four phylum-level clades, i.e., *Chloroflexota*, *Deinococcota, Bacteroidota*, and *Armatimonadota* were relatively highly abundant (7–32% of total reads). Indeed, they have been often detected as majority groups in moderately hot springs at circumneutral pH (e.g., Miller et al., 2009; Portillo et al., 2009; Song et al., 2013; Wang et al., 2013; Uribe-Lorio et al., 2019) similar to our sampling site. In contrast, archaeal members were minor (~1% of total reads) in the community. Eukaryotic 18S rRNA gene sequences were also rarely detected (<1%).

### Metagenome-assembled genomes

To assess the metabolic potential of each member in the community, we constructed a total of 130 MAGs (>20% completeness, <10% contamination) from the assembly (Supplementary Table S3). Of the 130 MAGs, 14 were classified as cMAGs consisting of single circular contigs, 40 were HQ MAGs, 46 were medium-quality (MQ)-MAGs, and 30 were low-quality (LQ)-MAGs, based on minimum information about metagenome-assembled genome (MIMAG) as previously defined (Bowers et al., 2017). Of the 13,365 contigs in the assembly, 2,337 contigs were binned into the 130 MAGs (Supplementary Table S3; Supplementary Figure S5).

Of the 46 MQ MAGs, three MAGs (mg025, mg055, and mg058) met the minimal standards for HQ MAGs, except for the presence of the 5S rRNA gene. In addition, one MAG (mg001) classified in *Nanoarchaeota* and three MAGs (mg089, mg092, and mg094) classified in *Patescibacteria* showed relatively low completeness values of 65.8–73.6% with the contamination value of 0%, although these MAGs consisted of ≤5 contigs and contained 16S and 23S rRNA genes. Such features have been already reported in most genomes of *Nanoarchaeota* and *Patescibacteria* (Castelle et al., 2018). In this study, the above seven MQ MAGs were exceptionally treated as “HQ MAGs.”

**Figure 2:**
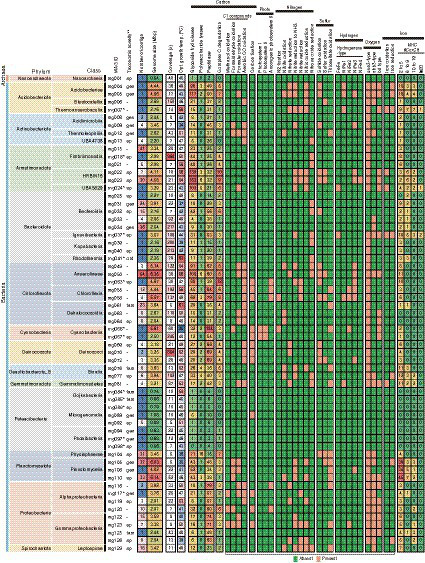
Summary of general feature and metabolic potential for the 61 HQ MAGs. Taxonomy, number of contigs, genome size, coverage, estimated optimum growth temp, and gene context for main metabolism are shown. *IDs of cMAGs are in bold. **Taxonomic novelty: the novelty of taxonomic ranks of our MAGs against the latest GTDB R207: ord, order level; fam, family level; gen, genus level; species level.

A total of the above 61 HQ MAGs (including 14 cMAGs, 40 “standard” HQ MAGs, and seven “additional” HQ MAGs) were used for the following genomic characterization. The general features of the 61 HQ MAGs were summarized in Figure 2 (Supplementary Table S3 for details). The genome size of the MAGs varied between 0.56 and 6.35 Mbp, which was in direct proportion to the number of predicted CDSs (*r*^2^ = 0.97, Supplementary Figure S6), as reported for genomes of cultivated isolates (Konstantinidis and Tiedje, 2004). The AAI among the 61 HQ MAGs ranged from 43.5 to 89.5% (Supplementary Table S4), indicating that each MAG differed from the others atleast the species level (Konstantinidis et al., 2017). About 47 of the 61 HQ MAGs, including 14 cMAGs, consisted of <10 contigs.

The GTDB-based taxonomic classification indicated that the 61 HQ MAGs were classified into diverse taxa, i.e., one archaeal and 13 bacterial phylum-level clades (Figure 2), which included the abundant taxa, such as *Chloroflexota*, *Deinococcota, Bacteroidota*, and *Armatimonadota*, in the microbial community as shown by the read-based analysis (Figure 1). Regarding the read coverages, a MAG (mg018) of *Armatimonadota* showed the highest value (864×), followed by mg070 of *Deinococcota* (854×), mg067 of *Cyanobacteria* (245×), and mg034 of *Bacteroidota* (217×). Four MAGs (mg049, mg055, mg058, and mg063) of *Chloroflexota* showed over 100× read coverages. The trends in abundant taxa were consistent between read-based and MAG-based analyses.

Notably, nine of the 14 cMAGs were the first reported cMAGs for the family- to class-level clades that these MAGs belonged to; for example, a MAG (mg024) was the first reported cMAG in the class-level clade “c__UBA5829” of *Armatimonadota*. In addition, of the 61 HQ MAGs, 1, 5, 13, and 24 MAGs were novel at order-, family-, genus-, and species-levels among MAGs in the latest GTDB R207.

#### Physiological potential

Predicted optimal growth temperatures (OGTs) of bacteria and archaea represented by the MAGs ranged from 25 to 63°C (Figure 2). Of the 61 HQ MAGs, 20 were estimated to be derived from thermophiles with 50°C or higher predicted OGTs. This result is consistent with the moderate high temperature (52°C) of the hot spring environment. Indeed, 19 MAGs belonged to thermophiles-containing clades, such as *Thermoanaerobaculia* of *Acidobacteriota*, *Thermoleophia* of *Actinobacteriota*, *Fimbriimonadia* of *Armatimonadota*, and *Rhodothermia* of *Bacteroidota*. In contrast, OGTs of some MAGs were predicted to be 25–40°C, suggesting that these MAGs were derived from mesophiles living at a lower temperature in this environment. Otherwise, they might represent thermotolerant microorganisms. Indeed, a cyanobacterial MAG (mg066; 25°C of the predicted OGT) was identified as *Fischerella thermalis,* which is a cosmopolitan species in hot spring environments and can grow at 15–58°C (Alcorta et al., 2019).

**Figure 3:**
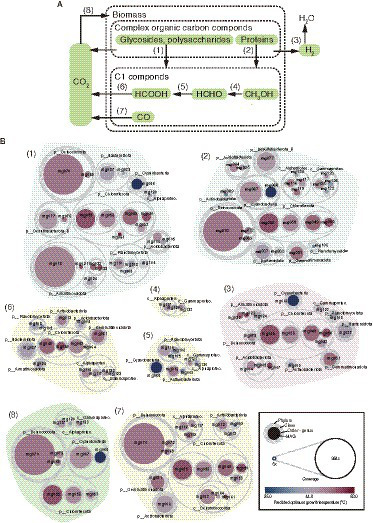
MAG-based model of carbon cycle. **(A)** Outline of carbon cycle and **(B)** the 61 HQ MAGs derived from prokaryotes potentially involved in each step (indicated by the numbers in parenthesis).

**Figure 4:**
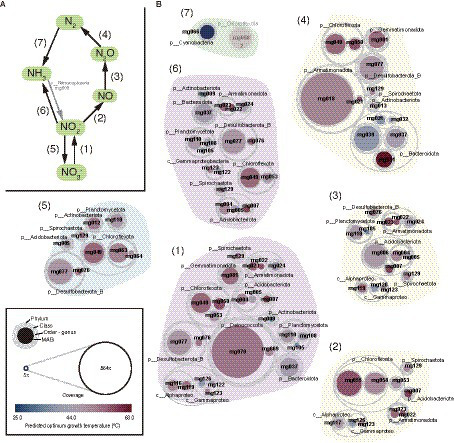
MAG-based model of nitrogen cycle. **(A)** Outline of nitrogen cycle and **(B)** the 61 HQ MAGs derived from prokaryotes potentially involved in each step (indicated by the numbers in parenthesis). It should be noted that ammonia oxidation to nitrite could be conducted by a *Nitrososphaeria* archaeon represented by a MQ MAG (mg003).

Gene context related to metabolism for each MAG is also summarized in Figure 2 (details are shown in Supplementary Table S5). In this paper, we focus on autotrophy (i.e., carbon fixation), phototrophy, degradation of organic carbon, and metabolisms of hydrogen, nitrogen, iron, and sulfur, which are commonly important for the ecosystem functioning of hot spring environments. Based on the predicted metabolism and abundance of the MAGs, we propose an overview of their functional roles in the biogeochemical cycles of carbon (Figure 3) and nitrogen (Figure 4). The predicted OGTs of MAGs suggest that the metabolic reactions occurred in a wide-range of temperatures by each microorganism. It should be noted that most genes for the biogeochemical cycling were lacking in the MAGs of *Nanoarchaeota* and *Patescibacteria* with small genome size (0.54–1.08 Mbp), and they might be symbionts to others, as reported previously (Castelle et al., 2018).

#### Carbon fixation

In the hot spring sediment, the main primary producers of this ecosystem are likely to be members of *Cyanobacteria* and *Chloroflexota* (Figure 3). High-coverage MAGs of *Cyanobacteria* and *Chloroflexota* encoded the Calvin–Bassham–Benson (CBB) and 3-hydroxypropionate pathways, respectively, for carbon fixation (Supplementary Table S5). Indeed, the two MAGs (mg066 and mg067) of *Cyanobacteria* encoded genes for photosystems I and II, and three MAG (mg053, mg055, and mg058) of *Chloroflexota* encoded genes for anoxygenic photosystem II. Thus, they are likely to use solar energy for carbon fixation. In addition, some of the low-coverage MAGs of *Alphaproteobacteria* and *Gammaproteobacteria* had genes for the CBB pathway, suggesting that they may also contribute to carbon fixation in this system.

*Meiothermus* MAGs of *Deinococcota* also encoded a key gene, ribulose 1,5-bisphosphate carboxylase/oxygenase (form I) of the CBB pathway (Supplementary Table S5), as previously reported in other *Meiothermus* spp. (Muller et al., 2016; Raposo et al., 2019). As described above, a *Meiothermus* MAG (mg070) was highly abundant among the MAGs. Although no autotrophs of *Meiothermus* spp. have been reported so far, the *Meiothermus* members represented by the MAGs are potentially involved in carbon fixation. If this is the case, the energy sources for carbon fixation by *Meiothermus* spp. might be carbon monoxide or sulfide based on their gene context.

One of the MAGs of *Patescibacteria* (mg089) encoded *aclAB* for ATP-citrate lyase of the reverse tricarboxylic acid (rTCA) cycle for carbon fixation, as reported in other *Patescibacteria* MAGs (Probst et al., 2017); however, as well as the previously-reported *Patescibacteria* MAGs, the mg089 MAG did not encode other genes for rTCA cycle, and thus, probably not represent an autotroph.

#### Organic carbon degradation

Complex organic carbon compounds, including carbohydrates and proteins, could be degraded by some bacterial members in the hot spring sediment (Figure 3). Relatively high numbers of genes (71 or higher, up to 153) for glycoside hydrolyses (GH) were detected in MAGs of *Acidobacteriae* of *Acidobacteriota*, the family-level clades (i.e., HRBIN16 and UBA5829) of *Armatimonadales*, *Ananerolineae* of *Chloroflexi*, and *Phycisphaerae* of *Planctomyces*. Some of these MAGs have also high numbers of genes (five or higher, up to 16) for polysaccharide lyases (PL). Indeed, the numbers of complex carbon degradation pathways (such as cellulose, hemicellulose, and chitin degrading pathways; Supplementary Table S5) were abundantly (six or higher) detected in these MAGs. Relatively high numbers of genes (40 or higher, up to 104) for peptidases were detected in taxonomically varied MAGs including *Cyanobacteria*, *Chloroflexia*, and *Anaerolineae* of *Chloroflexota*, *Alphaproteobacteria*, and *Gammaproteobacteria* of *Proteobacteria*, and *Deinococcota*. Some of the MAGs of *Cyanobacteria*, *Deinococcota*, and *Chloroflexota* were relatively highly abundant in the community.

One carbon (C1) compound from the complex organic carbon degradation could be sequentially used for some bacterial members as energy and carbon sources (Figure 3). Genes for oxidation of methanol and formaldehyde were found in relatively low abundant MAGs that were affiliated with *Alphaproteobacteria*, *Gammaproteobacteria*, *Actinobacteriota*, *Cyanobacteria*, and *Planctomycetota*. In contrast, genes for oxidation of formate and carbon monoxide were found in more diverse, higher abundant MAGs including those of *Acidobacteriota*, *Armatimonadota*, *Bacteroidota*, *Chloroflexota*, *Deinococcota,* and *Gemmatimonadota*.

#### Production and oxidation of hydrogen

The released electron from the degradation of organic carbon could be used for H_2_ production *via* fermentation, and sequentially, the produced H_2_ could be used by hydrogen-oxidizers as an energy source (Figure 3). Genes for hydrogenases involved in the production and oxidation of H_2_ were found in 18 out of the 61 HQ MAGs; five for *Chloroflexota*, each three for *Acidobacteriota* and *Armatimonadota*, and one each for *Actinobacteriota*, *Bacteroidota*, *Cyanobacteria*, *Deinococcota*, *Gemmatimonadota*, *Planctomycetota*, and *Gammaproteobacteria*. The detected hydrogenases included a variety of [FeFe]- and [NiFe]-hydrogenases (Figure 2; Supplementary Table S5), based on the HydDB classifier (Sondergaard et al., 2016). The detected [FeFe]-hydrogenases were classified in the groups A and C, which might be involved in H_2_-evolution and -sensing, respectively (Sondergaard et al., 2016). The detected [NiFe]-hydrogenases were classified in groups 1–4, which might be involved in respiratory H_2_-uptake, −sensing, and/or -evolution (Sondergaard et al., 2016). The above microorganisms represented by the MAGs potentially produce and/or oxidize H_2_.

#### Nitrogen cycle

Nitrate (NO_3_^−^) and other oxidized nitrogen species, i.e., nitrite (NO_2_^−^), nitric oxide (NO), and Nitrous oxide (N_2_O), could be used as electron acceptors by diverse microorganisms *via* denitrification or dissimilatory nitrate reduction to ammonia (DNRA; Figure 4). Genes for each step in denitrification/DNRA were found in taxonomically diverse MAGs (Figure 2). Based on the coverage-based abundance, members of the following taxonomic groups might be major players in each step: *Deinococcota* for NO_3_^−^ reduction to NO_2_^−^, *Chloroflexota* for NO_2_^−^ reduction to NO, *Chloroflexota* and *Desulfobacterota*_B for NO_2_^−^ reduction to NH_3_, *Acidobacteriota* for NO reduction to N_2_O, and *Armatimonadota* and *Bacteroidota* for N_2_O reduction to N_2_, respectively.

Regarding nitrification, the *nxr* genes for nitrite oxidation were found in MAGs of several phyla, such as *Chloroflexota* and *Desulfobacterota*_B. The *amo* genes for NH_3_ oxidation were found only in an archaeal MQ MAG (mg003) classified in the family *Nitrosocaldaceae* that includes NH_3_ oxidizers (Abby et al., 2018; Daebeler et al., 2018).

N_2_ could be fixed by a member of *Cyanobacteria*, of which the MAG (mg066) harbored *nifHDK* genes for nitrogenase, the key enzyme for nitrogen fixation. The mg066 MAG was classified in *Fischerella thermalis*, a thermophilic diazotroph (Alcorta et al., 2019), at the same species level. Although a *nifH* gene was found in a MAG (mg058) of *Chloroflexota*, no other *nif* genes were found. Therefore, it is doubtful that this bacterium represented by the MAG can fix N_2_.

#### Iron cycle

Another potential electron acceptor is ferric iron, i.e., Fe(III). Genes for MtrABC, which are involved in reduction of insoluble iron oxides (Shi et al., 2016; Deng et al., 2018), were found in five out of the 61 HQ MAGs (Figure 2); three for *Acidobacteriota*, and one each for *Desulfobacterota*_B and *Gammaproteobacteria*. These bacteria represented by the MAGs are potentially iron reducers. The Mtr subunits include multiheme *c*-type cytochromes (MHCs); for example, the MtrA and MtrC of iron-reducing *Shewanella* spp. represent decaheme *c*-type cytochromes. Genes for multiheme *c*-type cytochromes (MHCs) including Mtr were found in 49 HQ MAGs (Figure 2). In particular, those with 10 or more heme-binding motifs were found in 16 HQ MAGs, which were mostly corresponding to the above MAGs possessing the *Mtr* genes. For example, a circular MAG (mg007) classified as *Thermoanaerobaculum aquaticum*, which is an iron-reducer of *Acidobacteria* (Losey et al., 2013), encoded eight genes for MHCs with 10 or more heme-binding motifs, including one MtrC and two MtrA (Supplementary Table S6). Three MAGs (i.e., mg005, mg037, and mg105) possessing genes for MHCs that were predicted to be located on the outer membrane or extracellular space (Supplementary Table S6), suggesting that they could be involved in the reduction of solid Fe(III) oxides or in extracellular electron transfer from/to insoluble minerals. In contrast, no gene for PplA, which is a key protein in iron reduction by Gram-positive bacteria (Light et al., 2018, 2019), was found in any HQ, MQ, and LQ MAGs.

The reduced iron, i.e., ferrous iron (Fe^2+^), could be used by iron oxidizers as the electron donor, although its concentration was undetectable (<18 μM) at this site. Genes annotated as Cyc2, which are involved in iron oxidation by a variety of acidophilic and neutrophilic iron-oxidizing bacteria (Castelle et al., 2008; Kato et al., 2015; Garber et al., 2020), were found in four MAGs; each one for *Acidobacteriota* (mg007), *Armatimonadota* (mg022), *Bacteroidota* (mg037), and *Gemmatimonadota* (mg081). Phylogenetic analysis (Supplementary Figure S7 A) indicated that all the detected Cyc2 were classified into the cluster 3 as defined previously (McAllister et al., 2020). In addition, a gene annotated as MtoA, which is involved in iron oxidation by a neutrophilic iron-oxidizing bacterium *Sideroxydans lithotrophicus* (Liu et al., 2012), were found in a MAG (mg024) of *Armatimonadota*. MtoA is a homolog of the MtrA described above, that of PioA involved in iron oxidation by a phototrophic iron-oxidizing bacterium *Rhodopseudomonas palustris* (Jiao and Newman, 2007), and also that of DmsE involved in the reduction of dimethyl sulfoxide by *Shewanella oneidensis* (Gralnick et al., 2006). Phylogenetic analysis (Supplementary Figure S7 B) indicated that the MtoA of mg024 was relatively close to the PioA of *P. palustris* and MtoA of *Sideroxydans lithotrophicus* rather than MtrA and DmsE of *Shewanella* spp. Notably, all of the five MAGs (i.e., mg007, mg022, mg024, mg037, and mg081) also encoded genes for MHCs with 10 or more heme-binding motifs as described above. Thus, the bacteria represented by these MAGs are potentially capable of oxidation and/or reduction of iron at circumneutral pH.

#### Sulfur cycle

Considering the low salinity in this sediment environment, it is unlikely that sulfate is the major electron acceptor for microorganisms. Indeed, no genes for DsrAB, which is the key enzyme for sulfate reduction, were found in all HQ, MQ, and LQ MAGs, suggesting that there are no or few sulfate-reducing microorganisms. In contrast, several genes (for example, *sqr*/*fcc*, *sor*, and *sox*) for oxidation of reduced sulfur species, such as sulfide (HS^−^), elemental sulfur (S°), and thiosulfate (S_2_O_3_^2−^), were found in some MAGs. These microorganisms represented by the MAGs might use such reduced sulfur species, which could be produced by fermentation of organic sulfur compounds, as electron donors.

### Hot spring *Armatimonadota*

As described above, we successfully obtained six HQ MAGs including two cMAGs of the phylum *Armatimonadota* (Tamaki et al., 2011; Oren and Garrity, 2021) that has been formerly called OP10 (Hugenholtz et al., 1998). Members of this phylum are widely distributed in a broad range of environments, including hot springs, and contain phylogenetically diverse species (Lee et al., 2014). Despite the phylogenetic diversity, only four cultivated species have been reported so far as follows; one thermophilic species, i.e., *Chthonomonas calidirosea* (Lee et al., 2011), and three mesophilic species, i.e., *Capsulimonas corticalis* (Li J. et al., 2019), *Armatimonas rosea* (Tamaki et al., 2011), and *Fimbriimonas ginsengisoli* (Im et al., 2012). Although several MAGs of *Armatimonadota* have been obtained from high-temperature environments (Eloe-Fadrosh et al., 2016; Ward et al., 2017; Kato et al., 2018), their metabolic potential has been limitedly described. Therefore, little is known about the ecophysiology of the phylum *Armatimonadota*, especially thermophilic members.

#### Phylogeny

**Figure 5:**
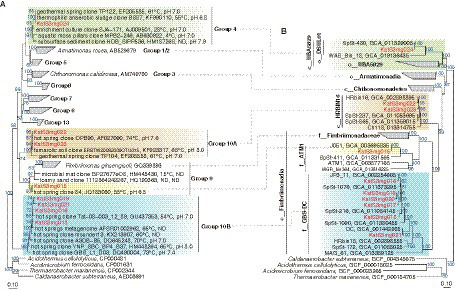
Phylogeny of *Armatimonadota*. **(A)** Phylogenetic tree of 16S rRNA genes and **(B)** Phylogenomic tree based on 120 bacterial marker proteins are shown. The MAGs obtained in this study are colored in red. Group numberings shown in the 16S rRNA gene tree **(A)** are originated from the previous report (Lee et al., 2013).

The *Armatimonadota* MAGs recovered in this study were classified in the class *Fimbriimonadia* (mg015, mg018, and mg021), and class-level clades of “c__HRBIN16” (mg022 and mg023) and “c__UBA5829” (mg024). In particular, the MAGs of *Fimbriimonadia* were classified in the family-level clades of “f__ATM1” (mg015) and “f__GBS-DC” (mg018 and mg021). The MAGs of mg018 and mg024 were the first reported cMAGs of “f__GBS-DC” and “c__UBA5829,” respectively. Phylogenetic trees for the *Armatimonadota* MAGs based on 16S rRNA genes and highly conversed marker proteins are shown in Figure 5, indicating that the genome-based clades of “f__ATM1,” “f__GBS-DC,” “c__HRBIN16,” and “c__UBA5829” are corresponding to the 16S rRNA gene-based clades (Lee et al., 2013) of Group 9, Group 10B, Group 10A, and Group 4, respectively.

#### Metabolic potential

**Figure 6:**
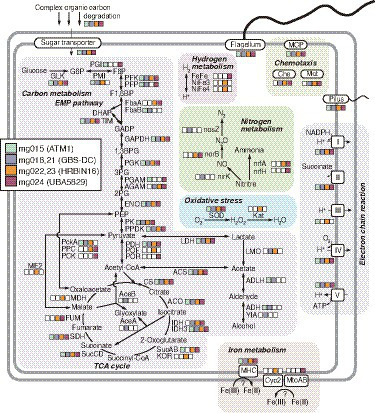
Metabolic potential for *Armatimonadota* members deduced from the HQ MAGs. Details of genes in the pathways are listed in Supplementary Table S7, in addition to Supplementary Tables S5, S6.

As described above (Figure 2), members represented by the *Armatimonadota* MAGs are likely to be aerobic chemoorganoheterotrophs. More details of the metabolic potential of the MAGs of each clade of the *Armatimonadota* are summarized in Figure 6 (Supplementary Table S7 for details). Sugars produced by the degradation of complex organic compounds can be transported into the cells *via* a variety of transporters, and used as their energy and carbon sources. The MAGs had a complete gene set of the Embden-Meyerhof-Parnas (EMP) pathway for glycolysis, suggesting the members represented by the MAGs degrade glucose to pyruvate, although some differences in the encoded genes for the EMP pathway were observed between the clades; for example, fructose-bisphosphate aldolase class II (FbaA) for “c__HRBIN16” and “c__UBA5829,” but Fba class I (FbaB) for the others, at the pathway from fructose 1,6-bisphosphate (F1,6BP) to dihydroxyacetone phosphate (DHAP) and glyceraldehyde 3-phosphate (GADP). The pyruvate could be oxidized to acetyl-CoA by pyruvate dehydrogenase (PDH) for all clades, by pyruvate-ferredoxin/flavodoxin oxidoreductase (POF) for “c__HRBIN16,” and by pyruvate:ferredoxin oxidoreductase (POR) for “c__UBA5829.” The acetyl-CoA could be degraded to organic acids *via* each step of the tricarboxylic acid (TCA) cycle. The MAGs of “c__HRBIN16” encoded a complete gene set for the TCA cycle, but the others lacked genes for malate dehydrogenase (MDH) and/or fumarate hydratase (FUM). Thus, only the members of “c__HRBIN16” could effectively produce reductants (e.g., NADPH and quinol) from the acetyl-CoA.

The reductants generated *via* the above EMP pathway and complete/incomplete TCA cycle could be used for ATP synthesis *via* aerobic respiration. Regarding oxidative stress, genes for superoxide dismutase (SOD) were found in all the clades, but those for catalase were found only in “c__HRBIN16.” All the MAGs encoded genes for pilus and flagellum, suggesting that they are motile. Indeed, genes for chemotaxis-related proteins (MCP, Che, and Mot) were also found in all the clades, except “f__ATM1.”

Notably, the presence of POR/POF, which generate reduced ferredoxin (Fd)/flavodoxins (Fld) generally found in anaerobes, strongly supports that the members represented by the MAGs of “c__HRBIN16” and “c__UBA5829” are facultative anaerobes. This is consistent with the result that only the MAGs of “c__HRBIN16” and “c__UBA5829” encoded genes for hydrogenase involved in H_2_-evolving fermentation using Fd/Fld, and those for MHCs with six or more heme-binding motifs for iron reduction. Moreover, as described above, genes for Cyc2 or MtoAB-like proteins were found in a MAG of “c__HRBIN16” (mg022) or that of “c__UBA5829” (mg024), respectively, implying that they can oxidize Fe(II) as the energy source.

#### Abundance and global distribution

As already described (Figure 1), the *Armatimonadota* members accounted for approximately 10% of the whole prokaryotic community in the hot spring sediment. To assess the relative abundance of each of the *Armatimonadota* members represented by the MAGs in the community, we counted HiFi reads containing CDSs for the single copy maker protein (i.e., RpsB or RplC; Supplementary Figure S8). The operational taxonomic unit (OTU) of RpsB or RplC corresponding to those in the MAG (mg018) at a 99% amino acid identity level showed the highest proportion among all *Armatimonadota* OTUs including unbinned sequences, as consistent with its highest coverage value among the *Armatimonadota* MAGs (and even among all the 130 MAGs; Figure 2). Considering its high abundance and metabolic potential as described above, this member represented by the mg018 MAG is likely to highly adapt to the environmental conditions at the hot spring sediment, and may play a significant role in carbon and nitrogen cycles. In contrast, the other MAGs were minor (Supplementary Figure S8), as consistent with their low coverage values (Figure 2). Even so, based on their metabolic potential, the members in “c__HRBIN16” and “c__UBA5829” may play a role in the degradation of complex carbon degradation, and biogeochemical cycles of iron and nitrogen in a different way from the abundant member from the mg018 MAG. In addition, hundreds of *Armatimonadota* OTUs consisting of unbinned sequences were detected in the metagenome, some of which were ranked in the top 10 abundant members (Supplementary Figure S8), indicating that there are more diverse *Armatimonadota* members in this environment, although their ecological roles are unclear.

To investigate where the members represented by the *Armatimonadota* MAGs are abundant on a global scale, we surveyed 16S rRNA genes closely related to those of our MAGs in public databases. We found that the *Armatimonadota* members have been relatively abundant (>2%, up to 19.5% of total reads) mainly in hot spring environments (41–90°C) in Japan, United States, China, India, and Antarctica, at circumneutral pH, in addition to some sludges and soils (Supplementary Table S8). As described above, the phylogenetic analysis (Figure 5) indicated that most of the close relatives to the *Armatimonadota* MAGs have been recovered from hot spring environments at circumneutral pH. Our study site fell into the ranges of temperature and pH where the close relatives have been detected. Thus, the members represented by the *Armatimonadota* MAGs are likely to be neutrophilic and moderate thermophilic, which is consistent with the predicted OGTs (43–56°C; Figure 2). The members of these *Armatimonadota* clades are likely to be globally distributed and relatively abundant in hot spring environments at moderately high temperatures (>40°C) and circumneutral pH, and may play a significant role in the biogeochemical cycling of carbon, nitrogen, and iron.

## Conclusion

In the present study, we retrieved the 61 HQ MAGs, including 14 cMAGs, of uncultivated bacteria and archaea from hot spring sediment (52°C) by PacBio long-read metagenomics. Notably, nine of the 14 cMAGs were the first reported cMAGs for the family- to class-level clades that these MAGs belonged to. The genome analysis suggested that these uncultivated prokaryotes play a significant role in the biogeochemical cycling of carbon, nitrogen, iron, and sulfur in this site. In particular, we showed that the members of *Armatimonadota*, which are widely distributed and frequently abundant in hot spring environments, might be aerobic, moderate thermophilic chemoorganoheterotrophs, and potentially oxidize and/or reduce iron. Our results expand the ecological potential of uncultivated bacteria in moderately-high-temperature environments. The genome information reported in this study will lead us to further cultivation and characterization that are needed to demonstrate the predicted metabolic function of the microbial dark matter.

## Data availability statement

The datasets used in this study can be found in the DDBJ under the accession numbers, DRA011957 and PRJDB11609. Nucleotide sequences of all contigs from the assembly are available in FigShare (DOI: 10.6084/m9.figshare.20447931).

## Author contributions

SK, KS, and MO conceived and designed this study. SK, SM, and AS performed the experiments and analyzed the data. SK, SM, KS, and MO wrote the manuscript. All authors contributed to the manuscript and approved the submitted version.

## Funding

This work was supported by Institute of Fermentation (IFO), JSPS KAKENHI Grant Number 19H05679, 19H05689, and 20H05592 (Post-Koch Ecology), 19H03310 and 22K19141, and the RIKEN interdisciplinary research program Integrated Symbiology (iSYM).

## Conflict of interest

The authors declare that the research was conducted in the absence of any commercial or financial relationships that could be construed as a potential conflict of interest.

## Publisher’s note

All claims expressed in this article are solely those of the authors and do not necessarily represent those of their affiliated organizations, or those of the publisher, the editors and the reviewers. Any product that may be evaluated in this article, or claim that may be made by its manufacturer, is not guaranteed or endorsed by the publisher.

## Footnotes

^1^ https://github.com/pacificbiosciences/unanimity/

^2^ https://github.com/tseemann/barrnap
