# Supplementary material for: Insights into ecological roles of uncultivated bacteria in Katase hot spring sediment from long-read metagenomics
Source: Front Microbiol. 2022 Nov 3;13:1045931. doi: 10.3389/fmicb.2022.1045931 (PMC9671151; doi:10.3389/fmicb.2022.1045931)
Supplement: Supplementary file 1 [file Data_Sheet_1.PDF]

Supplementary Material

1 Supplementary Data

All supplementary files are available on FigShare [DOI: 10.6084/m9.figshare.20447931]

2 Supplementary Figures and Tables

2.1 Supplementary Tables

Supplementary Table S1. List of 542 circular contigs (except 12 complete genomes)

Supplementary Table S2. Contigs of putative plasmids

Supplementary Table S3. Summary of the 130 MAGs obtained in this study

Supplementary Table S4. AAI values among HQ-MAGs

Supplementary Table S5. Gene context of the 130 MAGs

Supplementary Table S6. List of CDSs for MHCs

Supplementary Table S7. List of CDSs of *Armatimonadota* MAGs

Supplementary Table S8. Abundance of *Armatimonadota* 16S rRNA gene reads in other areas

2.2 Supplementary Figures

Supplementary Figure S1. Photo of the sampling site

Supplementary Figure S2. Assembly graph of the assembled metagenome

Supplementary Figure S3. Length, coverage, and GC content of contigs

Supplementary Figure S4. Categorization for circular contigs

Supplementary Figure S5. Size, coverage, and GC content of binned and unbinned contigs

Supplementary Figure S6. Genome size and number of CDSs of HQ-MAGs

Supplementary Figure S7. Phylogenetic trees of (A) Cyc2 and (B) MtoA

Supplementary Figure S8. Relative abundance of *Armatimonadota*

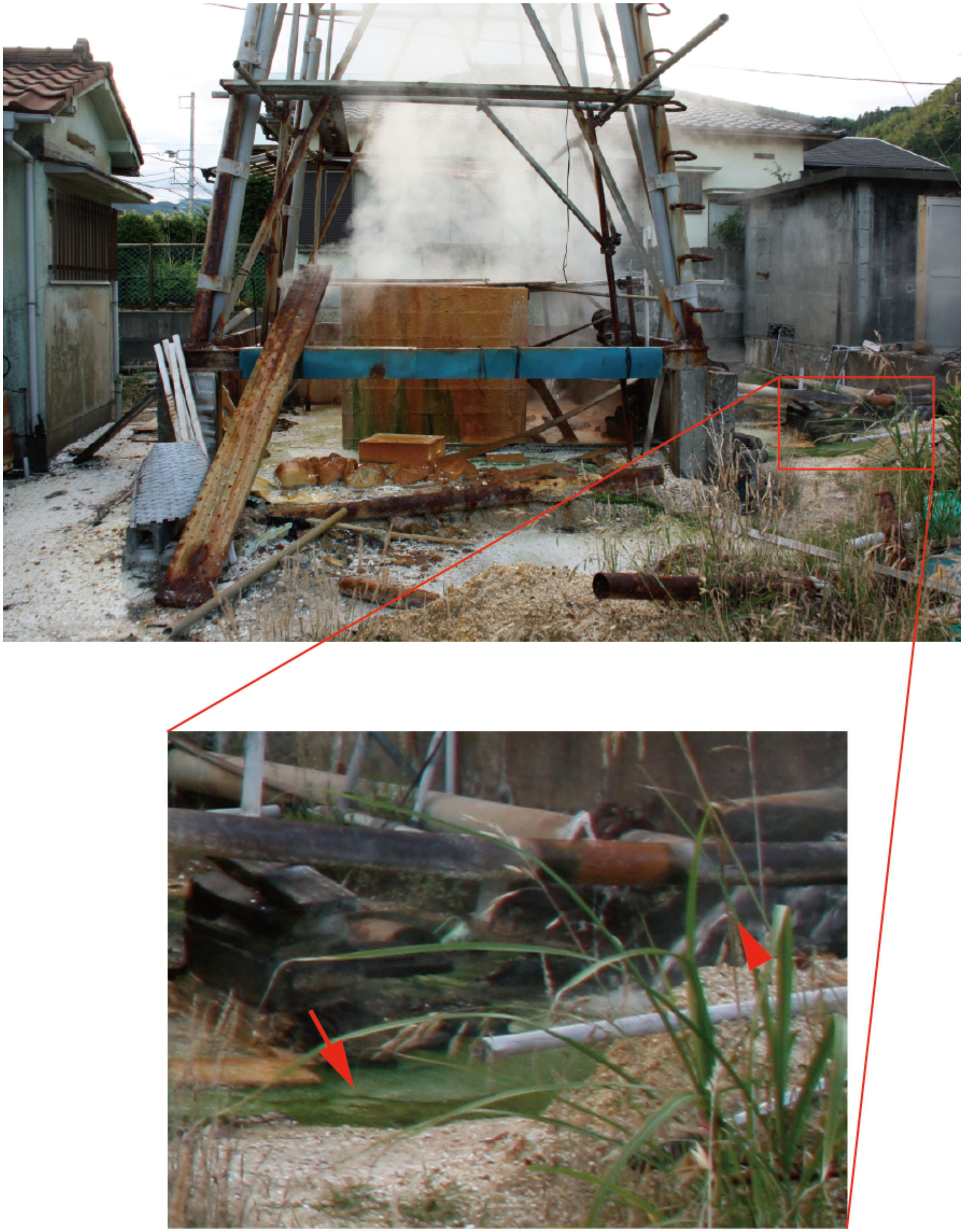

Supplementary Figure S1. Photo of the sampling site. The discharging point of hot spring water (84.5ºC; red arrowhead) and the sampling point for the sediment sample used in this study (52.1ºC; red arrow) are indicated.

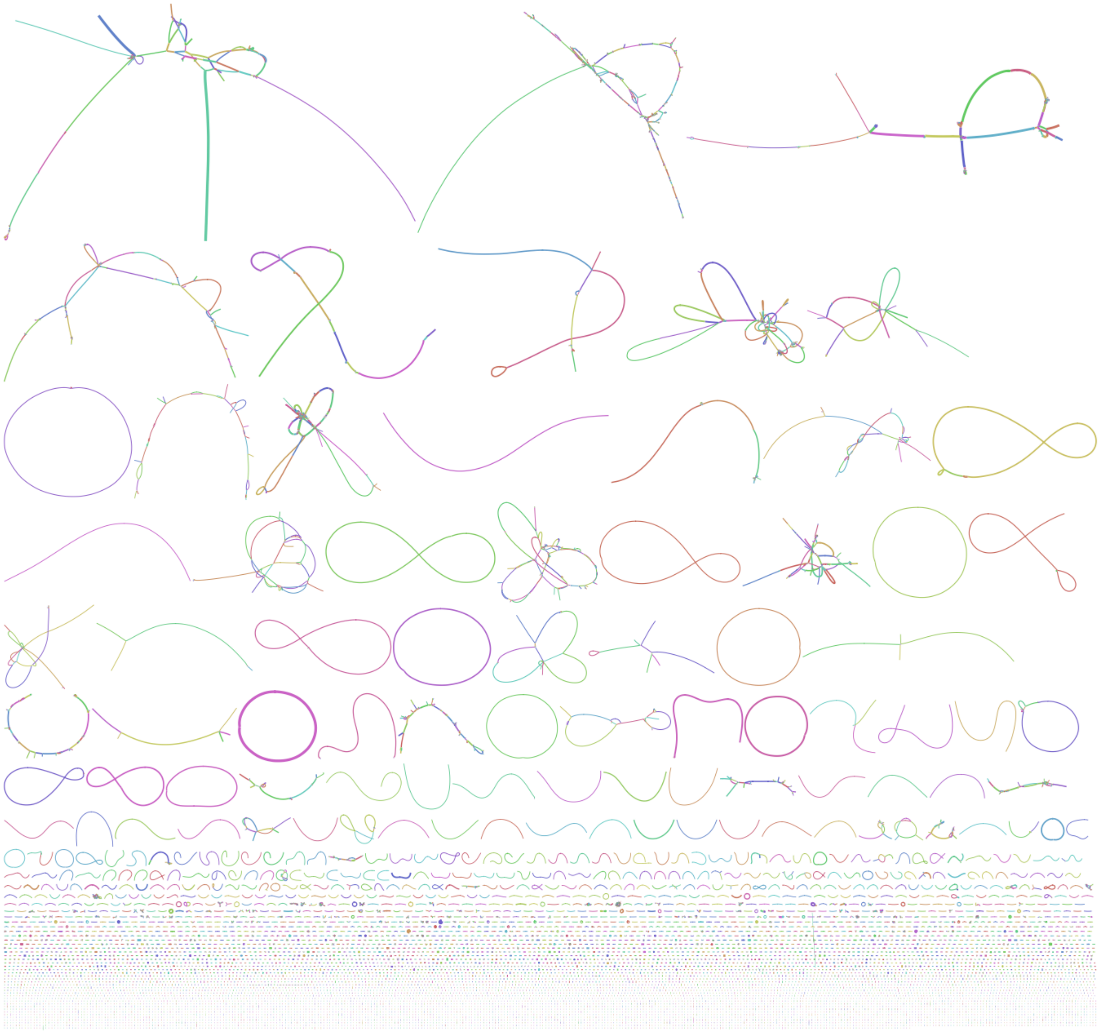

Supplementary Figure S2. Assembly graph of the assembled metagenome.

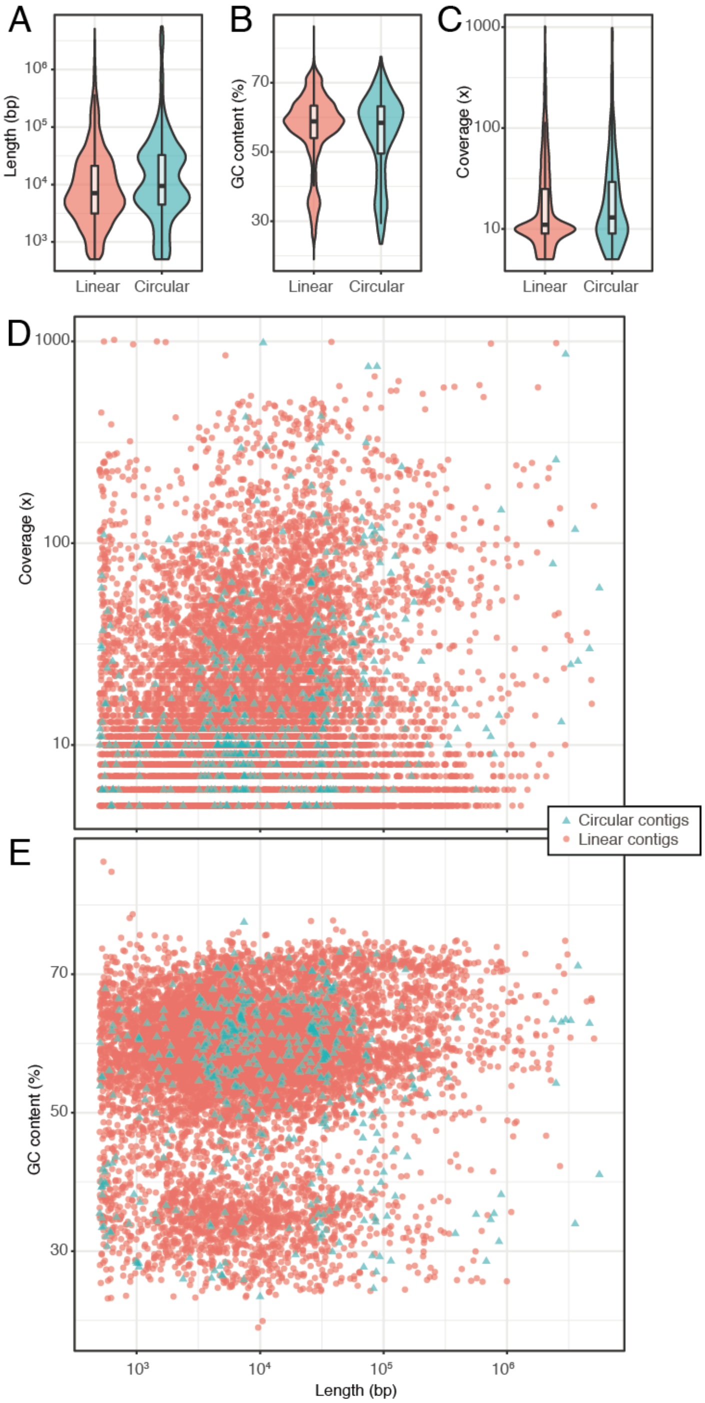

Supplementary Figure S3. Length, coverage, and GC content of contigs. Violin plots for distribution of **(A)** length, **(B)** GC content, and **(C)** coverage of linear and circular contigs are shown. Box plots are also shown within the violins. Scatter plots for contig length vs. **(D)** coverage, and vs. **(E)** GC content are shown. Circular (green) and linear (red) contigs are indicated.

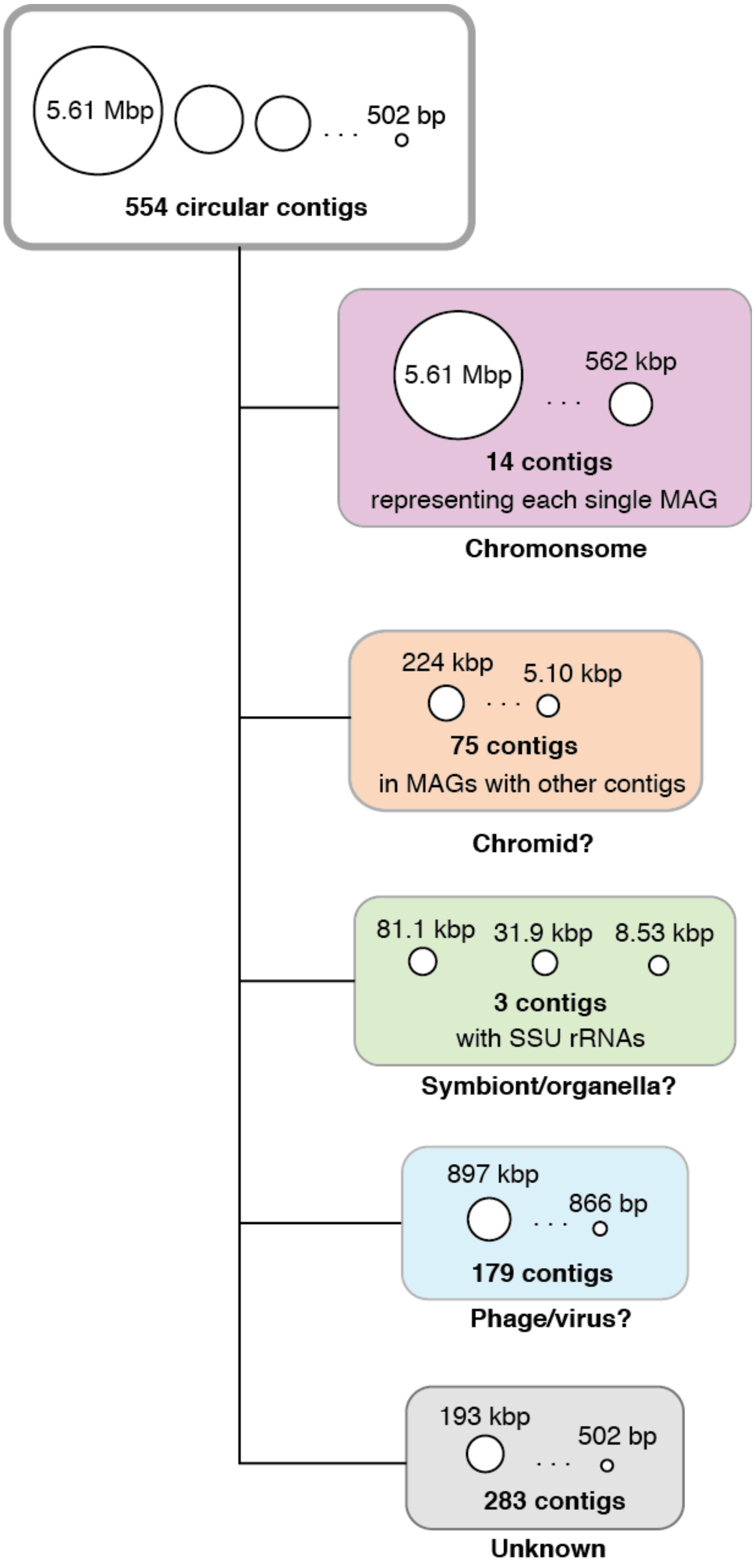

Supplementary Figure S4. Categorization for circular contigs.

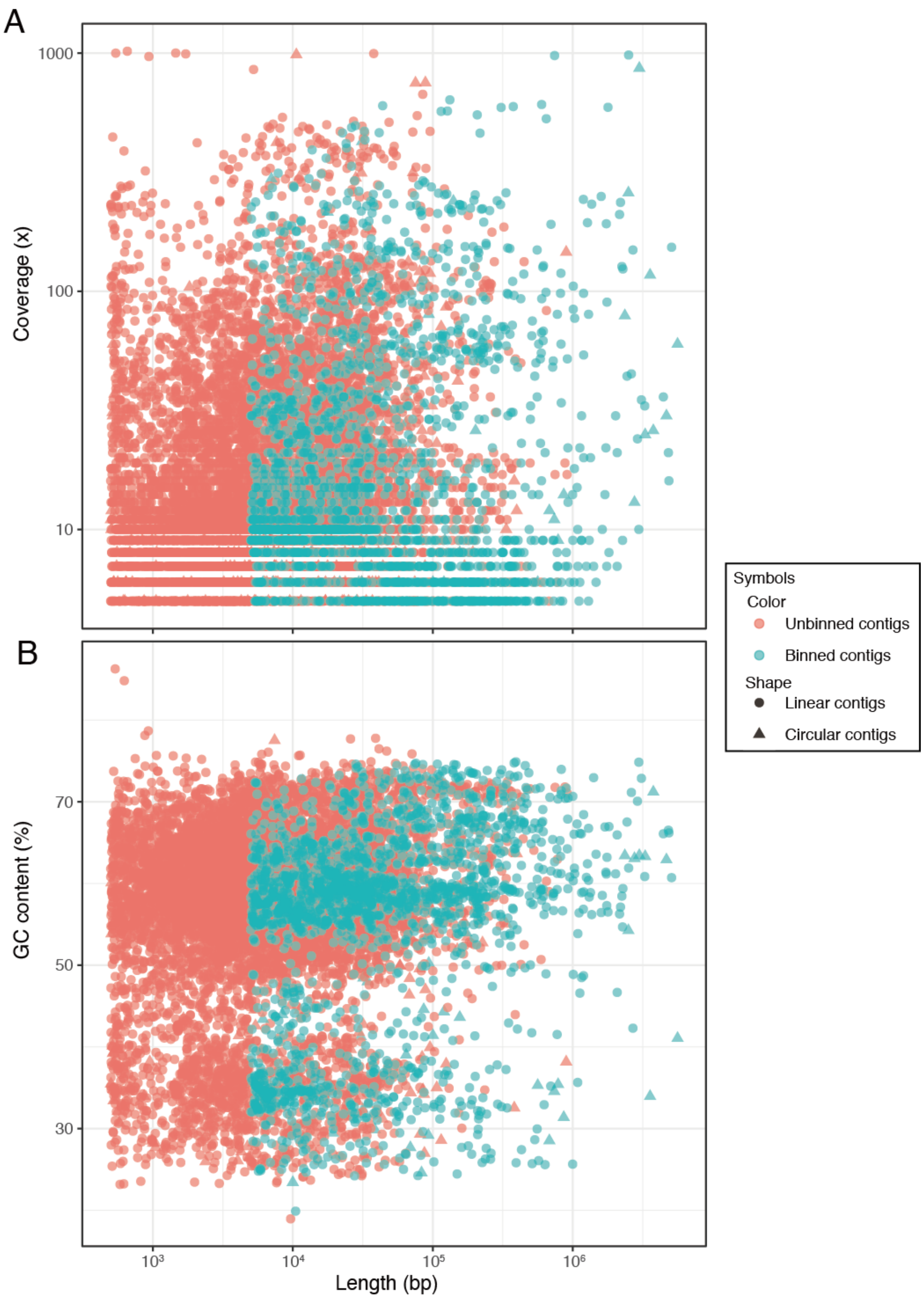

Supplementary Figure S5. Size, coverage, and GC content of binned and unbinned contigs. Plots for contig size vs. coverage **(A)** and vs. GC content **(B)** are shown. Contigs binned into all the MAGs including C-, HQ-, MQ-, and LQ-MAGs are colored in green.

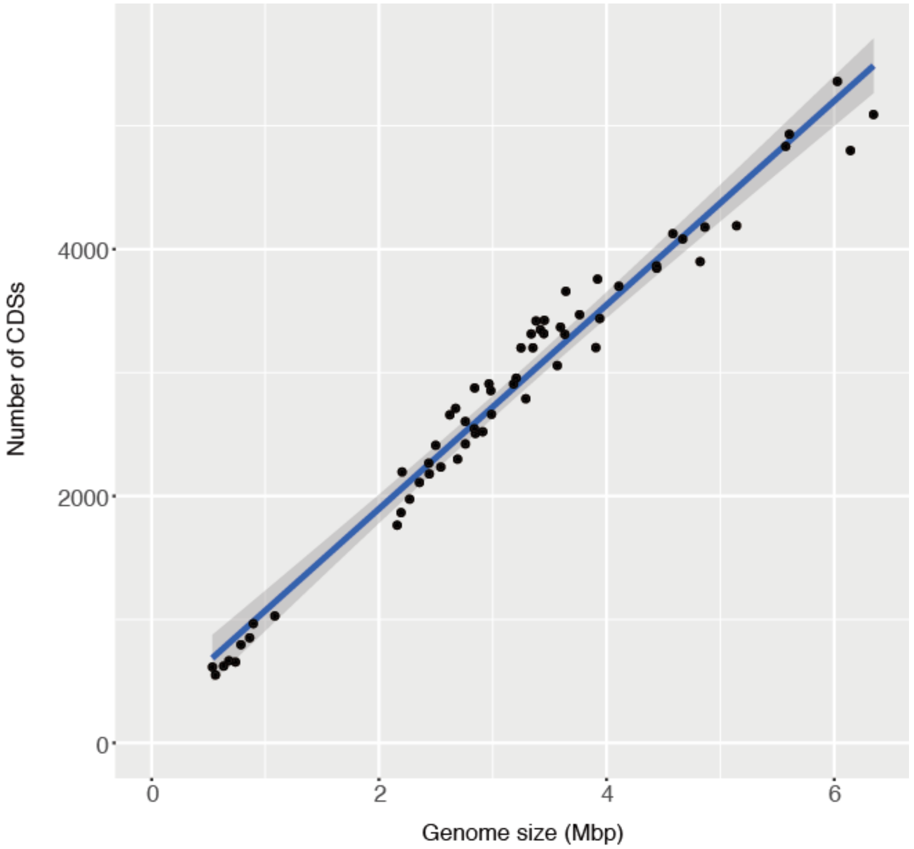

Supplementary Figure S6. Genome size and number of CDSs of HQ-MAGs. Regression line (blue line) with 99.9% confidence interval (dark-gray area) is shown.

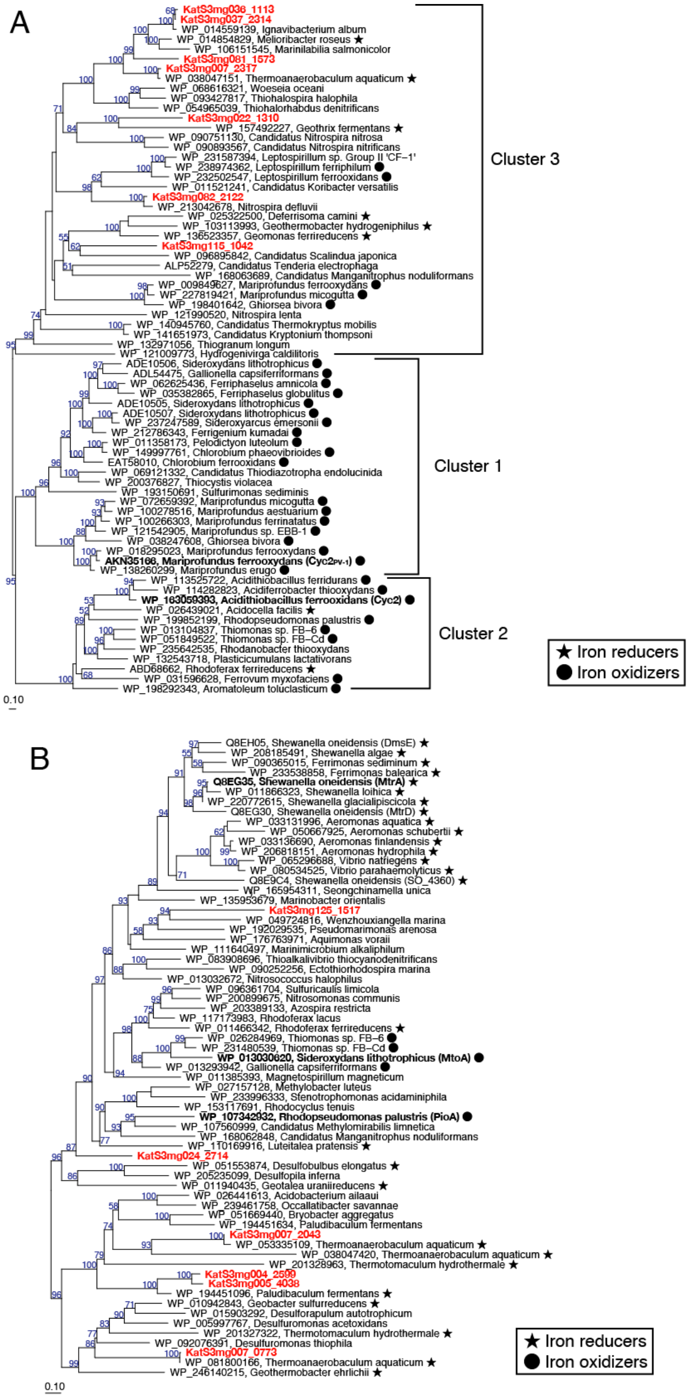

Supplementary Figure S7. Phylogenetic trees of (A) Cyc2 and (B) MtoA. The maximum-likelihood trees were constructed using IQ-TREE with the LG+I+G4 model. CDSs of the MAGs obtained in this study are colored in red. Bootstrap values (50% or higher) from 1000 replicates are shown at branching points.

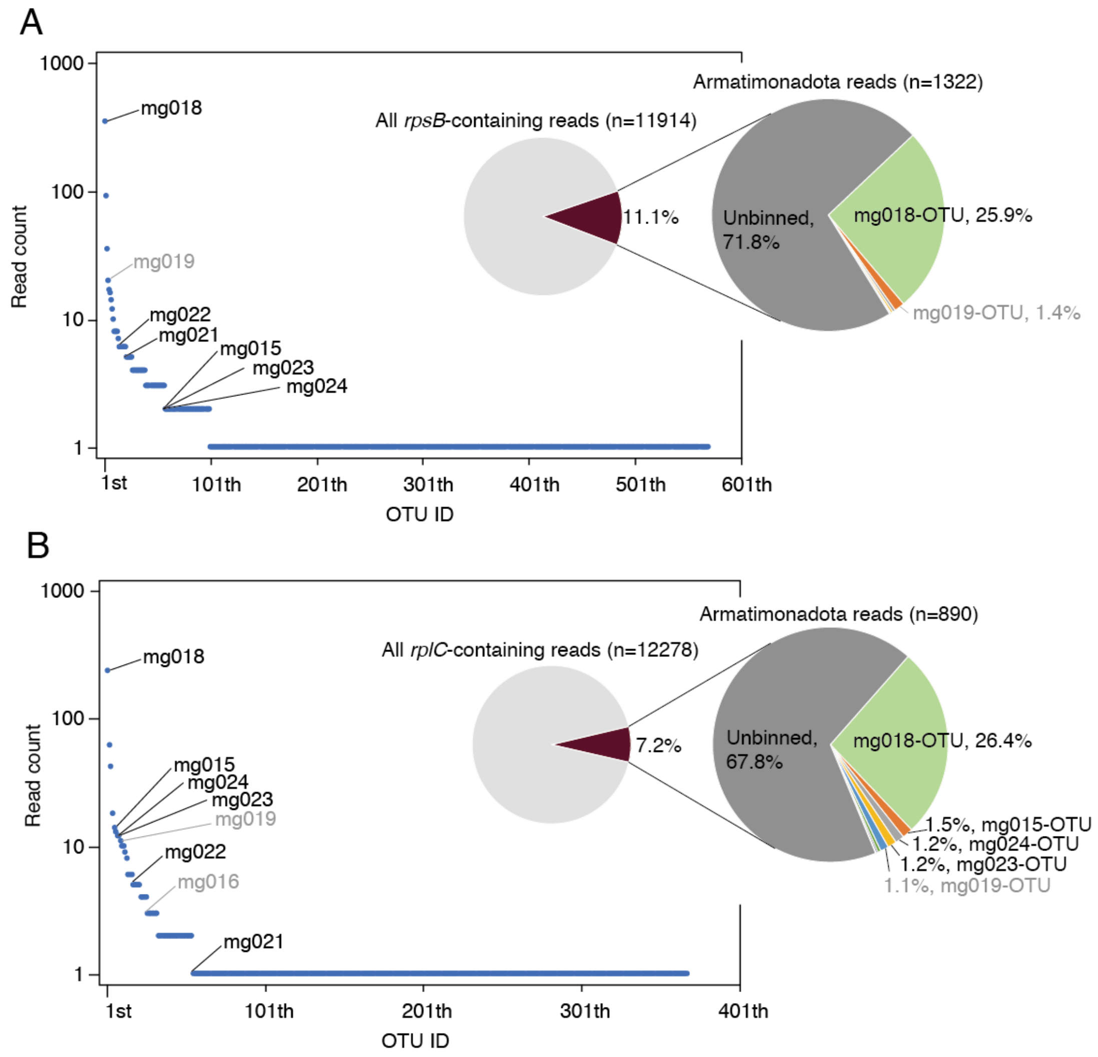

Supplementary Figure S8. Relative abundance of *Armatimonadota*. Rank abundance and proportion of *Armatimonadota* OTUs for (A) *rpsB* and (B) *rplC*-containing reads. In the rank abundance plots, OTUs (blue dots) corresponding to each CDS of HQ-MAGs (black letters) and of MQ- and LQ-MAGs (gray letters) are indicated.
